# Belonging in Practice: An Ethnographic Journey With a Clubhouse

**DOI:** 10.1093/geroni/igaf122.431

**Published:** 2025-12-31

**Authors:** Lydia Ogden

**Affiliations:** Salem State University, Salem, Massachusetts, United States

## Abstract

Older adults with serious mental illnesses (SMIs) face high rates of social isolation, loneliness, and associated health risks. However, psychosocial clubhouse membership offers protection through connection, community, and meaningful roles. From December 2023 to December 2024, I conducted a collaborative ethnographic study at a clubhouse with 35% older adult membership, spending 75 days in the field and conducting 40+ interviews. My research explores the clubhouse culture that fosters inclusion and belonging for this often-isolated population. Deeply participatory, the study prioritizes member voices through relationship-building, oral histories, and long-term engagement. Member-checking and collaborative analysis ensure alignment with lived experiences, and clubhouse members are invited as co-authors in final publications. This approach strengthens research trustworthiness while supporting local and national clubhouse policy. Findings will contribute to understanding how clubhouses sustain meaningful social connections and improve outcomes for older adults with SMIs.

